# En route to photoaffinity labeling of the bacterial lectin FimH

**DOI:** 10.3762/bjoc.6.91

**Published:** 2010-08-26

**Authors:** Thisbe K Lindhorst, Michaela Märten, Andreas Fuchs, Stefan D Knight

**Affiliations:** 1 Christiana Albertina University of Kiel, Otto Diels Institute of Organic Chemistry, Otto-Hahn-Platz 4, D-24098 Kiel, Germany, Fax: +49 431 8807410; 2 Swedish University of Agricultural Sciences, Department of Molecular Biology, Uppsala Biomedical Center, SE-75124 Uppsala, Sweden

**Keywords:** diazirines, FimH, lectins, MS/MS analysis, photoactive mannoside ligands, photoaffinity labeling

## Abstract

Mannose-specific adhesion of *Escherichia coli* bacteria to cell surfaces, the cause of various infections, is mediated by a fimbrial lectin, called FimH. X-ray studies have revealed a carbohydrate recognition domain (CRD) on FimH that can complex α-D-mannosides. However, as the precise nature of the ligand–receptor interactions in mannose-specific adhesion is not yet fully understood, it is of interest to identify carbohydrate recognition domains on the fimbrial lectin also in solution. Photoaffinity labeling serves as an appropriate methodology in this endeavour and hence biotin-labeled photoactive mannosides were designed and synthesized for photoaffinity labeling of FimH. So far, the photo-crosslinking properties of the new photoactive mannosides could be detailed with the peptide angiotensin II and labeling of FimH was shown both by MS/MS studies and by affino dot–blot analysis.

## Introduction

**Figure 1:**
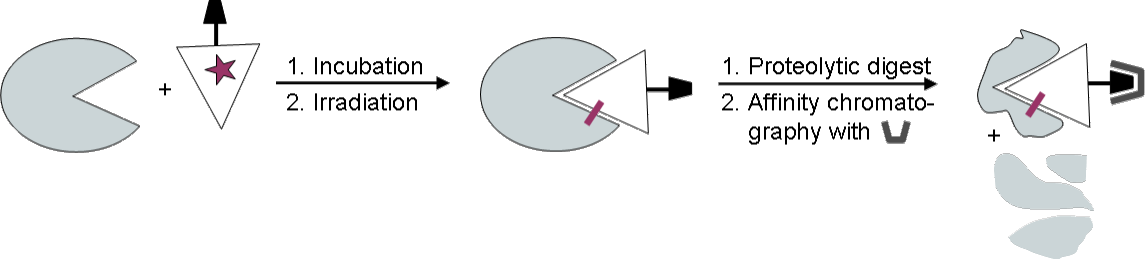
The photoaffinity technique allows the identification of ligand binding sites of a receptor protein after photo-crosslinking, proteolysis and affinity chromatography.

Photoaffinity labeling is a technique by which ligand binding sites of a receptor protein can be identified in solution. It requires a photoactive ligand derivative, which can form a reactive species upon photo-excitation. Thus, incubation of the photoprobe with a protein followed by irradiation can result in a photo-crosslinked product, that provides structural information on the binding site of the protein (Figure 1) [1].

It has become our goal to utilize this methodology for the investigation of carbohydrate binding of the bacterial lectin FimH. The protein FimH is found on the tips of type 1 fimbriae, long adhesive filamentous appendages on the surface of many bacteria, such as *Escherichia coli* [2–5]. In X-ray studies a carbohydrate recognition domain (CRD), which can complex one α-D-mannosyl residue, has been clearly identified [6–8]. However, other binding experiments performed with a multitude of synthetic as well as natural mannosides and oligomannosides are not in complete agreement with just one monovalent carbohydrate binding site on the FimH protein [9–13]. Thus, we decided to employ photoactive ligands to probe carbohydrate binding to the known CRD in solution and to identify possibly auxiliary, so far unknown, binding sites on bacterial lectin FimH [2].

**Figure 2:**
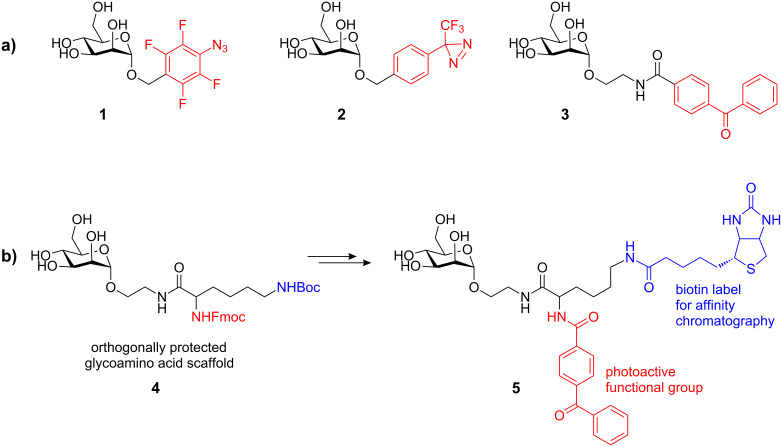
**a)** Three known photolabile α-D-mannosides that differ in the nature of the photoactive functional group (in red); **b)** the known bifunctional mannoside ligand **5** is based on the orthogonally protected glycoamino acid **4** and well suited for photoaffinity labeling.

The known FimH CRD was taken as the lead structure for the design of an appropriate photoactive ligand. Inspection of the available X-ray data clearly shows that α-D-mannosides are complexed in the CRD with the aglycone moiety sticking out of the binding pocket. The entrance of the CRD is flanked by the tyrosine residues Tyr48 and Tyr137 (‘tyrosine gate’) that can form favorable interactions with appropriate mannosidic aglycone moieties, such as π-π-stacking with the phenyl group of benzyl mannosides [14]. In a preceding work, we synthesized the three corresponding photoactive α-D-mannosides, **1**, **2**, and **3** (Figure 2a) for photolabeling of the bacterial lectin FimH [15]. They differ in their photoactive functional groups, which are part of the aglycone moiety. Upon irradiation, the aryl azide **1** and the diazirine **2** expel nitrogen to yield a reactive nitrene and a carbene intermediate, respectively. Irradiation of the benzophenone **3**, on the other hand, delivers a reactive triplet diradical.

In addition, in order to combine a photoactive functional group with an affinity label within the same mannoside, the orthogonally protected glycoamino acid scaffold **4** was synthesized and used for the preparation of the biotin-labeled photophore **5**, which is well suited for the streptavidine-based photoaffinity labeling, relying on the extraordinary high affinity of the protein streptavidine for biotin (Figure 2b) [16].

## Results and Discussion

In order to learn more about the ligand properties of the previously prepared photoactive mannosides **1**, **2**, **3**, and **5**, we have performed computer-aided docking studies using FlexX [17–19] to get an idea about their binding to the bacterial lectin FimH, as reported earlier [20]. FlexX produces so-called scoring values for each docked ligand, which can be regarded as a rough estimate of its free binding energy. Low (more negative) scores correlate with high affinities, whilst higher scores reflect diminished binding potency (Table 1). Docking was based on two different X-ray structures. In the first case, crystals with an open tyrosine gate were taken as the basis [7], whilst in the second case, a closed-gate conformation of FimH was used for the modeling [8]. This led to somewhat different predictions, as expected, however, the same trends were revealed for ligands **1**–**3** and **5** in comparison with the well-known standard ligands methyl α-D-mannoside (MeMan) and *p*-nitrophenyl α-D-mannoside (pNPMan), respectively (Table 1).

When these mannosides were tested as inhibitors of type 1 fimbriae-mediated bacterial adhesion to a mannan-coated surface in an ELISA [21–22], IC_50_-values were obtained, which reflect the concentration of the derivative employed, that leads to 50% inhibition of bacterial adhesion. Three independent measurements resulted in high standard deviations, a typical characteristic of this assay, whereas the relative trend in a series of tested ligands can be verified with high reproducibility. Hence, the inhibitory potencies of the tested ligands were referenced to an internal standard inhibitor, MeMan (inhibitory potency ≡ 1), to deduce relative inhibitory potencies, so-called RIP-values (Table 1). This uniform referencing shows, that all photoactive mannosides surpass the inhibitory potency of MeMan, and that mannosides **1** and **2** are more powerful inhibitors than **3** and **5**. Consequently, the synthetic photoactive mannosides are suited as ligands for the bacterial lectin FimH. Comparison of the measured IC_50_-values with the theoretical docking results, discloses a somewhat limited value of the computer-aided predictions in this case. Docking suggested that mannosides **2**, **3** and **5** are the most potent ligands of FimH in the tested series, which was not confirmed experimentally. On the other hand, it has to be kept in mind that the employed ELISA is not based on pure FimH, but rather on type 1-fimbriated bacteria, a dissimilar more complex system.

**Table 1:** Binding of mannoside ligands to FimH was predicted by computer-aided docking (FlexX) and measured by ELISA utilizing type 1-fimbriated *E. coli*. IC_50_-values are averaged from three independent experiments.

mannoside	FlexX Scores “open-gate”^a^	FlexX Scores “closed-gate”^b^	IC_50_ (SD)^c^ [mmolar]	RIP^d^

MeMan	−22.5	−23.3	1.84 (1.32)	1
pNPMan	−24.9	−27.4	0.04 (0.01)	46
**1**	−23.6	−24.0	0.06 (0.01)	31
**2**	−28.3^e^	−29.3^e^	0.12 (0.09)	15
**3**	−30.2	−29.8	0.20 (0.10)	9
**5**	−31.9	−36.0	0.63 (0.42)	3

^a^based on open-gate structure of FimH, PDB ID: 1KLF [7]. ^b^based on closed-gate structure of FimH, PDB ID: 1UWF [8]. ^c^SD: standard deviation. ^d^RIP: relative inhibitory potency. ^e^To facilitate docking, the diazirine ring was substituted by a cyclopropyl ring.

**Scheme 1:**
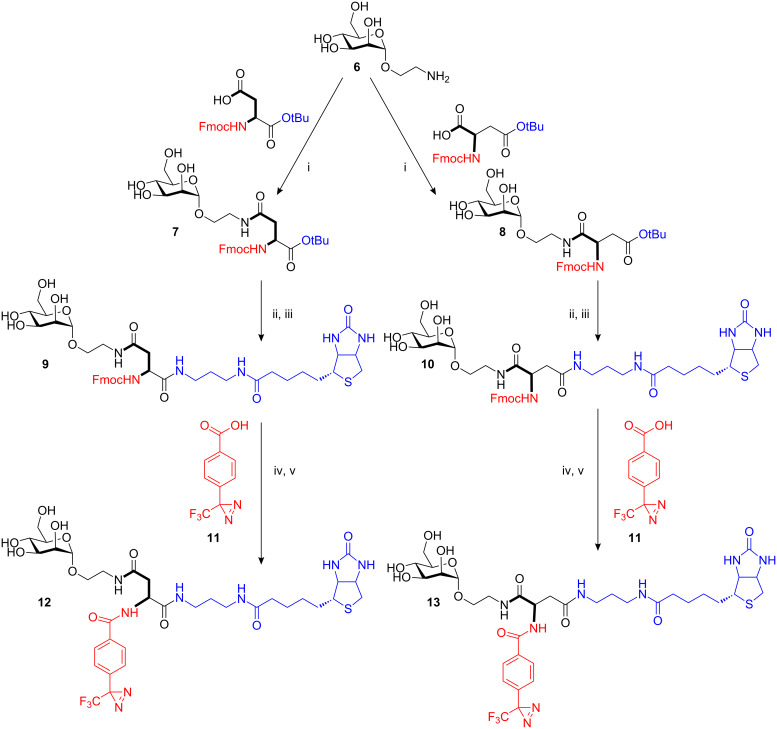
Synthesis of photoactive glycoamino acids **11** and **12**. i) Fmoc-Asp-O*t*Bu (for **7**), Fmoc-Asp(O*t*Bu)-OH (for **8**), HATU, DIPEA, dry DMF, N_2_, RT, 95% for **7** and 96% for **8**; ii) 80% aq TFA, RT; iii) (+)-biotinylamidopropylammonium trifluoroacetate, HATU, DIPEA, dry DMF, N_2_, RT, 48% (two steps) for **9** and 26% (two steps) for **10**; (iv) 20% piperidine, dry DMF, RT; v) HATU, DIPEA, dry DMF, Ar, RT, 81% (two steps) for **12** and 98% (two steps) for **13**.

The binding studies showed that mannosides with a typical photolabel in the aglycone moiety serve as ligands for FimH, and that even the more complex glycoamino acid derivative **5** is a suitable ligand. Encouraged by these results, we set out to improve the photochemical properties of **5** in order to facilitate later photoaffinity-labeling of FimH. Our earlier work suggested that diazirines are more useful photoactive groups than aryl azides and benzophenones [15]. Therefore, the synthesis of a biotin-labeled daizirine-functionalized mannoside was our next target. In this synthesis, aspartic acid was utilized as the scaffold molecule in two different ways in order to allow fine-tuning of the spacing between the mannoside ligand and the photoactive group. Thus, the amino acid derivatives Fmoc-Asp-O*t*Bu and Fmoc-Asp(O*t*Bu)-OH were employed in two analogous synthetic pathways, starting with peptide coupling to the known 2-aminoethyl mannoside **6** (Scheme 1) [23]. This led to the orthogonally protected mannoside amino acid *tert*-butyl esters **7** and **8**. The *tert*-butyl ester groups were then cleaved under acidic conditions and the resulting acids ligated with the biotin derivative biotinylamidopropylammonium trifluoroacetate. These two steps gave the Fmoc-protected biotin-labeled glycoamino acids **9** and **10**, respectively. Fmoc-cleavage and peptide coupling with the diazirine **11** led to the target molecules **12** and **13** in good yield.

**Scheme 2:**
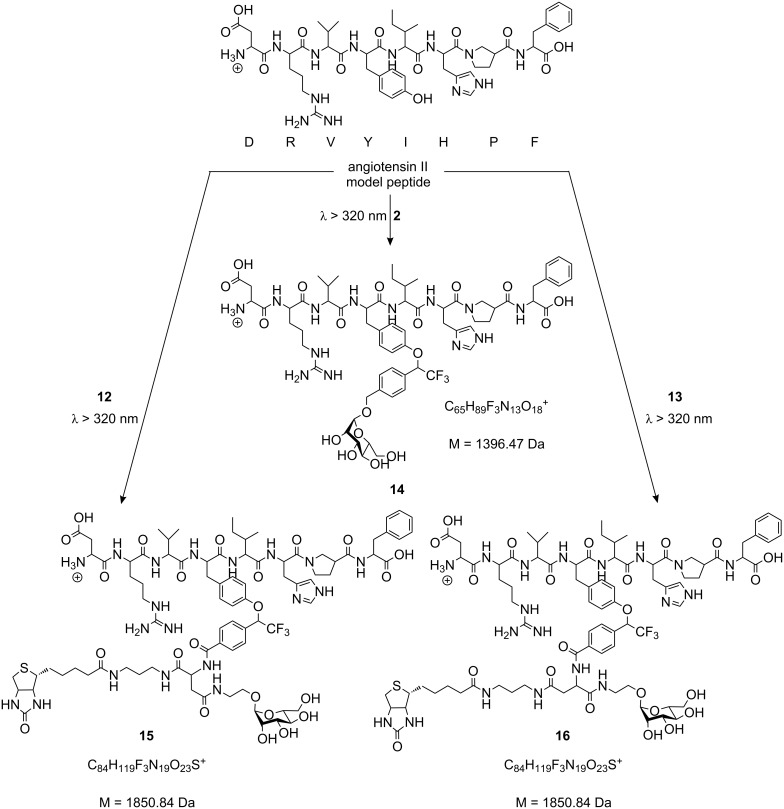
Photo-crosslinking experiments with the model peptide angiotensin II and the photoactive mannosides **2**, **12**, and **13**, respectively (see Experimental Part).

To test the prepared photoactive mannosides in crosslinking reactions, the model peptide angiotensin II (DRVYIHPF) was first employed. It was irradiated with the three different diazirine-functionalized mannosides **2**, **12**, and **13**. From our earlier work [15] it was known that irradiation of the diazirine-functionalized mannoside **2** with angiotensin II led to a photo-crosslinked product with *m/z* = 698.82. This double positively charged ion correlates with a 1:1-photoaddition product of peptide and photolabel with the molecular formula C_65_H_90_F_3_N_13_O_18_^2+^ and a monoisotopic mass of 1397.47 Da. We have now carried out MS/MS experiments to analyze the structure of this photo-crosslinked adduct and unequivocally shown that insertion of the carbene, which results after irradiation of **2**, occurs at the hydroxyl group in the side chain of the angiotensin tyrosine (Y) (see Experimental Part). This crosslinking reaction leads to the ether **14** (Scheme 2). Analogously, the photo-crosslinked products, obtained from irradiation of angiotensin II with either **12** or **13**, can be correlated with the structures of **15** and **16**, respectively, both showing a peak at *m/z* = 1851.1 in their mass spectra, corresponding to a molecular formula of C_84_H_119_F_3_N_19_O_23_S^+^ (monoisotopic mass M = 1850.84 Da).

The photo-crosslinking experiments with angiotensin II demonstrated, that the synthesized diazirines are well suited as photoprobes for the labeling of this peptide, with a preference for the tyrosine side chain. Thus, the photoactive mannosides were next investigated with the bacterial lectin FimH. For the irradiation experiments a FimH truncate, FimH_tr_, which resembles the adhesin domain of the complete FimH, was used [24]. FimH_tr_ comprises of amino acids 1-160 of FimH and is terminated by a histidine tag His_6_. FimH_tr_ has the same carbohydrate binding properties as FimH. Mass spectrometric analysis of FimH_tr_ revealed *m/z* = 17839 (calcd. *m/z* = 17845). Solutions of FimH_tr_ and the photophores **2**, **12**, and **13** were applied in a ratio of 5:1. Samples were incubated at 37 °C to allow formation of the lectin-ligand complex and then irradiated at λ ≥ 320 nm. This led to 1:1-photo-crosslinked products with the corresponding masses (Table 2).

**Table 2:** Results of mass spectrometric analysis of photo-crosslinked products, obtained from irradiation of FimH_tr_ with photoactive mannoside ligands **2**, **12**, and **13**.

FimH_tr_^a^ incubated with	FimH_tr_^b^ measured mass *m/z*	crosslinked product measured mass *m/z*	Δ mass *m/z* (FimH_tr_) - *m/z* (crosslinked product)	Δ mass expected

**2**	17858 [M + H]^+^	18200^c^ [M + H]^+^	342	350^e^
**12**	17905 [M + H]^+^	18672^d^ [M + H]^+^	767	804^f^
**13**	17697 [M + H]^+^	18500^d^ [M + H]^+^	803	804^f^

^a^For control experiments FimH_tr_ was irradiated similarly without addition of photophore. Mass spectrometric analysis revealed that the protein FimH survived the conditions of irradiation without any damage. ^b^Calcd. mass M = 17845 Da; measured values are acceptable within accuracy of the measurement. ^c^Measured on 4700 Proteomics Analyzer (Applied Biosystems). ^d^Measured on Bruker Biflex III. ^e^Based on the carbene resulting from irradiation of **2**: C_15_H_17_F_3_O_6_ (M = 350 Da); measured Δm-values acceptable within accuracy of the measurement. ^f^Based on the carbene resulting from irradiation of **12** and **13**, respectively: C_34_H_47_F_3_N_6_O_11_S (M = 804 Da); measured Δm-values acceptable within accuracy of the measurement.

**Figure 3:**
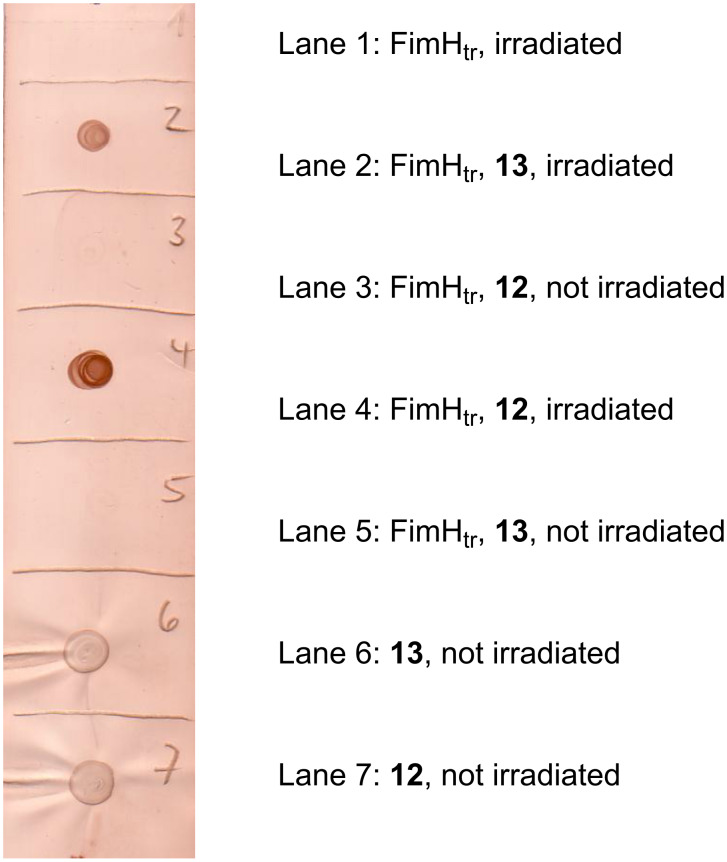
Affino dot–blot with FimH_tr_ and photoactive mannosides applied to nitrocellulose disks. It was irradiated, incubated with streptavidine–HRP conjugate and stained by addition of the chromogene 3,3’-diaminobenzidine. Photoaffinity probes **12** (lane 4) and **13** (lane 2) led to different color intensities, suggesting that ligand **12** is bound more tightly to FimH_tr_ than ligand **13**.

In addition to the mass spectrometric analysis, dot–blots were performed with FimH_tr_ and the photoactive mannosides. Affinity staining was carried out with a streptavidine–HRP conjugate and the chromogene 3,3’-diaminobenzidine (DAB). The biotin-labeled mannosides **12** and **13** gave violet spots on the nitrocellulose membrane when tested. Affino dot–blot with mannoside **2**, that does not contain a biotin moiety, was negative, as predicted. In addition, control experiments were carried out, leading to the expected results in all cases. Interestingly, photoaffinity probes **12** and **13** seem to exhibit unequal affinity to FimH_tr_ as suggested by the different intensity of color of the respective precipitates (Figure 3). Affinity staining using western blots led to analogous results (not shown).

## Conclusion

In conclusion, we have demonstrated the synthesis of new biotin-labeled photoactive mannosides for photoaffinity-labeling of the bacterial lectin FimH. The target molecules **12** and **13** were selected after docking studies based on the structure of FimH and according to binding studies employing type 1-fimbriated *E. coli*. Photo-crosslinking was tested with the model peptide angiotensin II and the regiochemistry of the insertion reaction could be solved by MS/MS studies. Furthermore, photoaffinity-labeling of FimH_tr_ was successful and could be demonstrated by mass spectrometric studies as well as dot–blot analysis.

The overall goal of this study is to identify mannose binding sites on the bacterial lectin FimH in solution. After photo-crosslinking of the lectin with photoactive, biotin-labeled mannosidic ligands, proteolytic digestion of the products of photo-crosslinking, followed by affinity chromatography and mass-spectrometric analysis of the fragments, should allow the identification of the critical amino acid residues on FimH, according to the photoaffinity methodology. However, so far we have not been successful in an unequivocal mass-spectrometric analysis of any proteolytic digest, we have obtained so far. Thus, this study is currently continued in our laboratory.

## Experimental

### Docking studies

Computer-aided modeling to predict binding of the various FimH ligands was carried out using FlexX flexible docking and consensus scoring as implemented in Sybyl 6.8 as described earlier [20]. Docking was based on published X-ray structures of the FimH CRD. This CRD was held fixed during the minimization, whereas the sugar ligand was allowed to change its conformation freely under the influence of the force field.

#### ELISA

ELISAs to determine IC_50_-values of the various FimH ligands were carried out with *E. coli* bacteria of strain HB101pPKL4 and mannan-coated microtiter plates as described earlier [21–22].

#### Mass spectrometry

**Figure 4:**
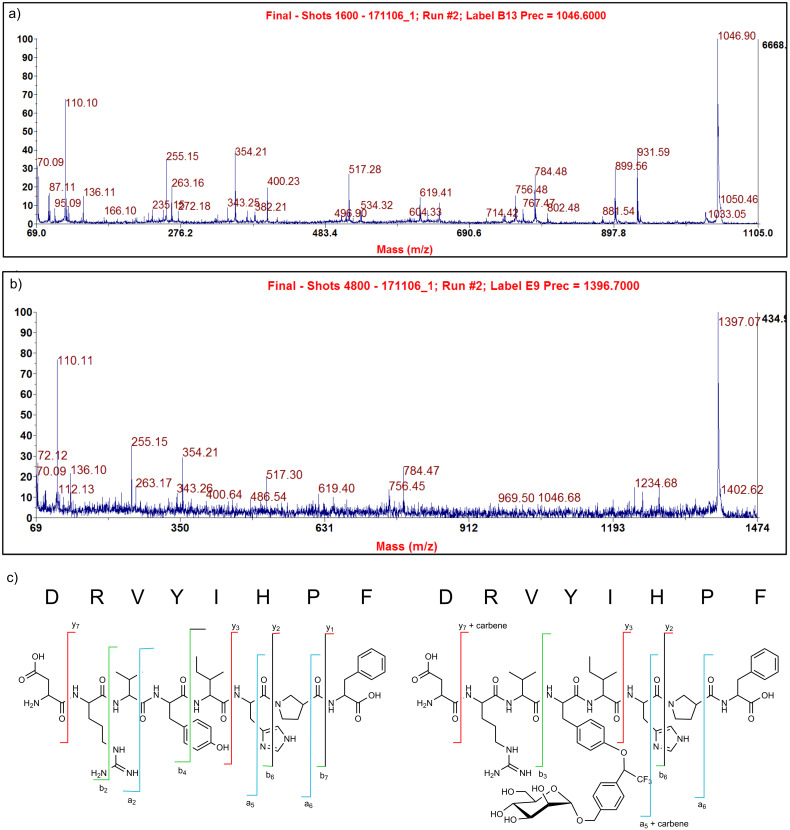
MS/MS spectra of angiotensin II (**a**) and of angiotensin II, photo-crosslinked with diazirine **2** (**b**), recorded on 4700 Proteomics Analyzer mass spectrometer (Applied Biosystems); (**c**) fragments of angiotensin II and photo-crosslinked angiotensin II according to Biemann’s nomenclature (Table 3).

For mass spectrometric analyses of the photo-crosslinked products, a Bruker Biflex III instrument (MALDI-TOF-MS, Prof. Th. K. Lindhorst, CAU), or a Bruker Biflex II instrument (ESI-FT-ICR-MS/MS, group of PD Dr. B. Lindner at the Research Center Borstel), or the 4700 Proteomics Analyzer mass spectrometer (Applied Biosystems MALDI-TOF/TOF-MS, group of Prof. M. Leippe, CAU) were used. Mass spectra were acquired using standard experimental sequences as provided by the manufacturer. Analysis of photo-crosslinking with angiotensin II is exemplified with diazirine **2** and presented in Figure 4.

**Table 3:** Assignment of peptide fragment ion pattern according to Biemann [25]; left two columns: angiotensin; right two columns photo-crosslinked product of angiotensin and diazirine **2** (Figure 4).

MS-MS experiment with angiotensin II *m/z*	corresponding fragment	photo-crosslinking of angiotensin II and 2; MS-MS experiment *m/z*	corresponding fragment

1046.90	[MH]^+^	1397.07	[MH]^+^
931.59	y_7_	1281.47	[MH – D]^+^
899.56	b_7_ – H_2_O	1046.68	[MH – carbene]^+^
881.54	b_7_	969.50	[a_5_ + carbene]
802.48	b_6_ – H_2_O		
784.48	b_6_	784.47	b_6_
767.47	b_6_ – NH_3_	756.45	a_6_
756.48	a_6_	619.40	a_5_
619.41	a_5_	517.30	b_4_ – NH_3_
534.32	b_4_	486.54	[Y + carbene]
517.28	b_4_ – NH_3_	400.64	y_3_
400.23	y_3_		
354.21	b_3_ – NH_3_	354.21	b_3_ – NH_3_
343.25	a_3_	343.26	a_3_
272.18	b_2_		
263.16	y_2_	263.17	y_2_
255.15	b_2_ – NH_3_	255.15	b_2_ – NH_3_
235.12	HP		
166.10	y_1_		
136.11	Y	136.10	Y
110.10	H	110.11	H
87.11	R	72.12	V
70.09	P	70.01	P

#### FimH truncate

The amino acid sequence of the FimH truncate [24], FimH_tr_, used in the photoaffinity labeling studies is as follows:

FACKTANGT AIPIGGGSAN VYVNLAPVVN VGQNLVVDLS TQIFCHNDYP ETITDYVTLQ RGSAYGGVLS NFSGTVKYSG SSYPFPTTSE TPRVVYNSRT DKPWPVALYL TPVSSAGGVA IKAGSLIAVL ILRQTNNYNS DDFQFVWNIY ANNDVVVPT GGHHHHHH

#### Affino dot–blot

Samples (2 μL, 1.15 mmolar) were applied on a nitrocellulose membrane, incubated with streptavidine-HRP conjugate and stained with 3,3’-diaminobenzidine.

#### Methods and materials for synthesis

Reactions were monitored by TLC on silica gel GF_254_ (Merck) with detection under UV light and by charring with 10% sulfuric acid in ethanol or using anisaldehyde and subsequent heating. Flash column chromatography was performed on silica gel 60 (40–63 µm, Merck) and for RP-MPLC a Merck Licroprep RP-18 column (Büchi) was used. Preparative HPLC was accomplished on a Shimadzu LC-8a machine (LiChrosorb RP-8, HIBAR). NMR spectra were recorded on Bruker AMX 400, Bruker DRX 500 or Bruker Avance 600 instruments. Chemical shifts are relative to TMS or the solvent peaks of CDCl_3_ (7.24 ppm for ^1^H, 77.0 ppm for ^13^C) or MeOD (3.35 ppm and 4.78 ppm for ^1^H, 49.3 ppm for ^13^C). Where necessary, assignments were based on 2D experiments (COSY, HSQC, HMBC or NOESY). IR spectra were taken with a Perkin Elmer FT IR Paragon 1000 (KBr). Optical rotations were measured with a Perkin-Elmer polarimeter (22 °C, 589 nm, length of cuvette: 1 dm). For MS analysis of the synthetic products MALDI-TOF mass spectra were measured with a Bruker Biflex III with 19 kV acceleration voltage. 4-Hydroxy-α-cyanocinnamic acid (HCCA) was used as matrix, either as a saturated solution in a solvent mixture (33% MeCN/ double distilled water and 0.1% TFA) or as saturated solution in acetone. Ionisation was effected with a nitrogen laser at 337 nm. ESI-MS spectra of the synthesized derivatives were measured with an Applied Biosystems Mariner ESI-TOF 5280 and millipore C_18_-pipette tips were used for ZipTipping^®^.

#### *N*-(Fluoren-9-ylmethoxycarbonyl)-4-[2-(α-D-mannopyranosyloxy)ethylamido]-L-aspartic acid *tert*-butyl ester (7)

The aminoethyl mannoside **6** (200 mg, 0.90 mmol), HATU (320 mg, 0.90 mmol) and Fmoc-Asp-O*t*Bu (400 mg, 0.98 mmol) were dried under vacuum for 10 min and then dissolved in dry DMF (12 mL) under a nitrogen atmosphere. DIPEA (300 μL, 2.25 mmol) was added and the reaction mixture stirred for 18 h at RT. The solvent was removed in vacuo and the residue purified by flash chromatography (ethyl acetate:MeOH:H_2_O = 6:2:1). Pure product fractions were pooled, filtered, concentrated and the residual taken up in water. Lyophylisation gave the title compound (531 mg, 0.86 mmol, 95%); *R*_f_ = 0.86 (ethyl acetate:MeOH:H_2_O = 6:2:1).

^1^H NMR (600 MHz, D_4_-MeOH): δ = 7.84 (d, 2H, *J* = 7.6 Hz, aryl-Fmoc), 7.71 (d, 2H *J* = 7.5 Hz, aryl-Fmoc), 7.43 (t, 2H, *J* = 7.5 Hz, aryl-Fmoc), 7.35 (t, 2H, *J* = 7.5 Hz, aryl-Fmoc), 4.81 (d, 1H, *J* = 1.5 Hz, H-1man), 4.50 (dd, 1H, *J* = 7.2 Hz, *J* = 5.6 Hz, Hα-asp), 4.40 (dd, 1H, *J* = 10.5 Hz, *J* = 7.2 Hz, Fmoc-CH*H*), 4.35 (dd, 1H, *J* = 10.5 Hz, *J* = 7.0 Hz, Fmoc-C*H*H), 4.28 (t, 1H, *J* = 7.0 Hz, Fmoc-C*H*-CH_2_), 3.88 (dd, 1H, *J* = 11.6 Hz, *J* = 2.2 Hz, manOCH_2_CH*H*), 3.87 (dd, 1H, *J* = 3.5 Hz, *J* = 1.8 Hz, H-2man), 3.79 (dd, 1H, *J* = 10.5 Hz, *J* = 4.5 Hz, manOCH*H*CH_2_), 3.75 (dd, 1H, *J* = 11.7 Hz, *J* = 5.9 Hz, manOCH_2_C*H*H), 3.74 (dd, 1H, *J* = 9.3 Hz, *J* = 3.4 Hz, H-3man), 3.65 (t, 1H, *J* = 9.5 Hz, H-4man), 3.58 (ddd, 1H, *J* = 9.7 Hz, *J* = 5.7 Hz, *J* = 2.1 Hz, H-5man), 3.57 (dd, 1H, *J* = 10.2 Hz, *J* = 4.3 Hz, manOC*H*HCH_2_), 3.47 (dd, 1H, *J* = 14.2 Hz, *J* = 6.2 Hz, H-6man), 3.04 (dd, 1H, *J* = 14.2 Hz, *J* = 4.7 Hz, H-6’man), 2.78 (dd, 1H, *J* = 15.1 Hz, *J* = 5.4 Hz, Hβ-asp), 2.69 (dd, 1H, *J* = 15.2 Hz, *J* = 7.3 Hz, Hβ’-asp), 1.49 (s, 9H, O*t*Bu) ppm; ^13^C NMR (150.9 MHz, D_4_-MeOH): δ = 170.92 (Fmoc-C=O), 170.63 (O*t*Bu-C=O), 156.92 (C-γ), 143.81, 141.15, 127.40, 126.79, 124.88, 119.54 (aryl-Fmoc), 100.28 (C-1man), 81.63 (O*C*(CH_3_)_3_), 74.78 (C-5man), 72.53 (C-3man), 72.05 (C-2man), 68.64 (C-4man), 68.12 (Fmoc-CH_2_), 67.17 (manO*C*H_2_CH_2_), 66.71 (manOCH_2_*C*H_2_), 51.63 (C-α), 48.30 (CH-Fmoc), 40.35 (C-6), 38.55 (C-β), 28.21 (OC(*C*H_3_)_3_) ppm.

MALDI-TOF-MS: *m/z* = 639.2 [M + Na]^+^, 656.2 [M + K]^+^; ESI-MS: *m/z* = 639.26 [M + Na]^+^ (616.66 calcd. for C_31_H_40_N_2_O_11_).

#### *N*-(Fluoren-9-ylmethoxycarbonyl)-4-(*tert*-butyl ester)-L-aspartic acid [2-(α-D-mannopyranosyloxy)ethyl]-amide (8)

The aminoethyl mannoside **6** (200 mg, 0.90 mmol), HATU (320 mg, 0.90 mmol) and Fmoc- Fmoc-Asp(O*t*Bu)-OH (400 mg, 0.98 mmol) were dried under vacuum for 10 min and then dissolved in dry DMF (12 mL) under a nitrogen atmosphere. DIPEA (300 μL, 2.25 mmol) was added and the reaction mixture stirred for 18 h at RT. The solvent was removed in vacuo and the residue purified by flash chromatography (ethyl acetate:MeOH:H_2_O = 6:2:1). Pure product fractions were pooled, filtered, concentrated and the residue taken up in water. Lyophylisation gave the title compound (536 mg, 0.87 mmol, 96%); *R*_f_ = 0.75 (ethyl acetate:MeOH:H_2_O = 6:2:1).

^1^H NMR (500 MHz, D_4_-MeOH): δ = 7.82 (dd, 2H, *J* = 7.6 Hz, *J* = 3.5 Hz, aryl-Fmoc), 7.71 (t, 2H, *J* = 7.7 Hz, aryl-Fmoc), 7.42 (dd, 2H, *J* = 7.5 Hz, *J* = 3.4 Hz, aryl-Fmoc), 7.34 (dd, 2H, *J* = 6.9 Hz, *J* = 1.1 Hz, aryl-Fmoc), 4.81 (d, 1H, *J* = 1.4 Hz, H-1man), 4.52 (dd, 1H, *J* = 8.7 Hz, *J* = 5.2 Hz, Hα-asp), 4.48 (dd, 1H, *J* = 10.5 Hz, *J* = 7.0 Hz, Fmoc-CH*H*), 4.39 (dd, 1H, *J* = 10.7 Hz, *J* = 6.6 Hz, Fmoc-C*H*H), 4.27 (t, 1H, *J* = 7.0 Hz, Fmoc-CH), 3.88 (dd, 1H, *J* = 12.1 Hz, *J* = 2.2 Hz, manOCH_2_CH*H*), 3.86 (dd, 1H, *J* = 1.4 Hz, *J* = 3.0 Hz, H-2man), 3.79 (dd, 1H, *J* = 10.7 Hz, *J* = 6.4 Hz, manOC*H*HCH_2_), 3.73 (dd, 1H, *J* = 8.8 Hz, *J* = 3.0 Hz, H-3man), 3.75 (dd, 1H, *J* = 11.8 Hz, *J* = 5.8 Hz, manOCH_2_C*H*H) 3.65 (t, 1H, *J* = 9.6 Hz, H-4man), 3.57 (ddd, 1H, *J* = 10.3 Hz, *J* = 7.1 Hz, *J* = 4.7 Hz, H-5man), 3.56 (dd, 1H, *J* = 10.8 Hz, *J* = 5.8 Hz, manOCH*H*CH_2_), 3.48 (dd, 1H, *J* = 13.9 Hz, *J* = 7.6 Hz, H-6man), 3.42 (dd, 1H, *J* = 13.9 Hz, *J* = 5.0 Hz, H-6’man), 2.82 (dd, 1H, *J* = 16.1 Hz, *J* = 5.1 Hz, Hβ-asp), 2.60 (dd, 1H, *J* = 16.1 Hz, *J* = 8.9 Hz, Hβ’-asp), 1.47 (s, 9H, OC(CH_3_)_3_) ppm; ^13^C NMR (150.92 MHz, D_4_-MeOH): δ = 173.48 (O*t*Bu-C=O), 172.48 (Fmoc-C=O), 171.39 (NH-(C=O)_asp_), 145.22, 142.59, 128.79, 128.17, 126.23, 120.91 (aryl-Fmoc), 101.64 (C-1man), 82.43 (O*C*(CH_3_)_3_), 74.67 (C-5man), 72.52 (C-3man), 72.05 (C-2man), 68.67 (C-4man), 68.19 (Fmoc-CH_2_), 67.06 (manO*C*H_2_CH_2_), 62.84 (manOCH_2_*C*H_2_), 53.32 (C-α), 48.34 (CH-Fmoc), 40.42 (C-6man), 38.74 (C-β), 28.33 (C(*C*H_3_)_3_) ppm.

MALDI-TOF-MS: *m/z* = 639.2 [M + Na]^+^; ESI-MS: *m/z* = 639.26 [M + Na]^+^ (616.66 calcd. for C_31_H_40_N_2_O_11_).

#### *N*-(Fluoren-9-ylmethoxycarbonyl)-4-[2-(α-D-mannopyranosyloxy)ethyl]-1-[(+)-biotinylamidopropyl]-L-aspartic acid diamide (9)

The glycoamino acid **7** (220 mg, 0.36 mmol) was dissolved in 80% aq TFA (5 mL) and stirred at RT for 60 min to cleave the *tert*-butyl ester. When the deprotection reaction was complete (TLC control in ethyl acetate:MeOH:H_2_O = 6:2:1) the solvent was removed in vacuo and the residue suspended in MeOH, filtered and the filtrate concentrated under reduced pressure. The resulting crude product was combined with HATU (150 mg, 0.43 mmol), (+)-biotinylamidopropylammonium trifluoroacetate (170 mg, 0.41 mmol) and dried for 30 min under vacuum. DMF (5 mL) and DIPEA (350 μL, 2.63 mmol) were then added under a nitrogen atmosphere. The reaction mixture was stirred overnight at RT, the solvent removed in vacuo and the residue purified by HPLC (MeCN-H_2_O gradient: 0–5 min 100% H_2_O, 5–20 min 60% H_2_O, 20–30 min 40% H_2_O, 30–40 min 0% H_2_O, 40–50 min 0% H_2_O, 50–60 min 100% H_2_O). The title compound was obtained after lyophylisation (145 mg, 0.17 mmol, 48%); *R*_f_ = 0.47 (ethyl acetate:MeOH:H_2_O = 6:2:1).

^1^H NMR (500 MHz, CD_3_CN:D_2_O = 1:1): δ = 7.85 (d, 2H, *J* = 7.6 Hz, aryl-Fmoc), 7.71 (dd, 2H, *J =* 6.8 Hz, *J* = 4.2 Hz, aryl-Fmoc), 7.45 (t, 2H, *J* = 7.4 Hz, Fmoc-H), 7.37 (t, 2H, *J* = 7.5 Hz, *J* = 4.1 Hz, Fmoc-H), 4.80 (d, 1H, *J* = 1.6 Hz, H-1man), 4.51 (dd, 1H, *J* = 7.6 Hz, *J* = 5.8 Hz, biotin-NHC*H*CH_2_S), 4.42 (dd, 1H, *J* = 11.8 Hz, *J* = 6.8 Hz, manOC*H*HCH_2_), 4.38 (dd, 1H, *J* = 11.8 Hz, *J* = 7.3 Hz, manOCH*H*CH_2_), 4.27 (d, 1H, *J* = 7.2 Hz, biotin-NHC*H*CHalkyl), 4.24 (t, 1H, *J* = 6.8 Hz, Hα-asp), 3.86 (dd, 1H, *J* = 3.4 Hz, *J* = 1.7 Hz, H-2man), 3.77 (dd, 1H, *J* = 12.1 Hz, *J =* 2.2 Hz, H-6man), 3.69 (dd, 1H, *J* = 9.6 Hz, *J* = 3.9 Hz. H-3man), 3.68 (dd, 1H, *J* = 12.2 Hz, *J* = 3.6 Hz, H-6’man), 3.65 (dd, 1H, *J* = 11.9 Hz, *J* = 6.9 Hz, Fmoc-CH*H*), 3.59 (t, 1H, *J* = 9.6 Hz, H-4man), 3.53 (ddd, 1H, *J* = 9.9 Hz, *J* = 5.5 Hz, *J* = 2.2 Hz, H-5man), 3.50 (dd, 1H, *J* = 11.2 Hz, *J* = 6.7 Hz, Fmoc-CH*H*), 3.41 (dd, 1H, *J* = 14.1 Hz, *J =* 5.3 Hz, manOCH_2_CH*H*), 3.28 (ddd, 1H, *J* = 14.2 Hz, *J* = 5.7 Hz, manOCH_2_C*H*H), 3.16 (dd, 1H, *J* = 9.3 Hz, *J* = 6.9 Hz, NH(C=O)C*H*H-CH_2_-CH_2_-CH_2_-biotin), 3.15 (t, 1H, *J* = 7.4 Hz, biotin-NHCHC*H*alkyl), 3.12 (dd, 1H, *J* = 9.5 Hz, *J* = 6.6 Hz, NH(C=O)CH*H*-CH_2_-CH_2_-CH_2_-biotin), 2.87 (dd, 1H, *J* = 12.8 Hz, *J* = 5.1 Hz, biotin-NHCHC*H*HS), 2.69 (dd, 1H, *J* = 12.9 Hz, *J* = 4.9 Hz, biotin-NHCHCH*H*S), 2.67 (dd, 1H, *J* = 13.7 Hz, *J* = 7.5 Hz, HN-C*H*H-CH_2_-CH_2_-NH), 2.59 (dd, 1H, *J* = 13.9 Hz, *J* = 7.8 Hz, HN-CH*H*-CH_2_-CH_2_-NH), 2.56 (dd, 1H, *J* = 14.2 Hz, *J* = 7.2 Hz, HN-CH_2_-CH_2_-C*H*H-NH), 2.53 (dd, 1H, *J* = 14.0 Hz, *J* = 6.8 Hz, HN-CH_2_-CH_2_-CH*H*-NH), 2.15 (t, 1H, *J* = 7.3 Hz, CH-Fmoc), 2.13 (d, 1H, *J* = 6.8 Hz, Hβ-asp), 2.11 (d, 1H, *J* = 6.5 Hz, Hβ’-asp), 1.66 (dd, 1H, *J* = 14.3 Hz, *J* = 7.6 Hz, NH(C=O)CH_2_-CH_2_-CH_2_-C*H*H-biotin), 1.64 (dd, 1H, *J* = 13.9 Hz, *J* = 7.0 Hz, HN-CH_2_-C*H*H-CH_2_-NH), 1.62 (dd, 1H, *J* = 14.0 Hz, *J* = 7.2 Hz, HN-CH_2_-CH*H*-CH_2_-NH), 1.59 (dd, 1H, *J* = 14.5 Hz, *J* = 7.0 Hz, NH(C=O)CH_2_-CH_2_-CH_2_-CH*H*-biotin), 1.54 (dd, 1H, *J* = 15.3 Hz, *J* = 8.0 Hz, NH(C=O)CH_2_-CH_2_-C*H*H-CH_2_-biotin), 1.49 (dd, 1H, *J* = 15.3 Hz, *J* = 8.1 Hz, NH(C=O)CH_2_-CH_2_-CH*H*-CH_2_-biotin), 1.33 (dd, 1H, *J* = 15.2 Hz, *J* = 7.5 Hz, NH(C=O)CH_2_-C*H*H-CH_2_-CH_2_-biotin), 1.28 (dd, 1H, *J* = 15.0 Hz, *J* = 7.3 Hz, NH(C=O)CH_2_-CH*H*-CH_2_-CH_2_-biotin) ppm; ^13^C NMR (125.75 MHz; CD_3_CN:D_2_O = 1:1): δ = 180.86 (C-γ), 176.52 (C_asp_(O)-NH-propyl), 173.26 (C_biotin_(O)-NH-propyl), 172.17 ((NH)_2_C=O), 165.62 (Fmoc-C=O), 144.81, 142.0, 128.93, 128.36, 126.25 (aryl-Fmoc), 100.73 (C-1man), 73.82 (C-5man), 71.68 (C-2man), 71.12 (C-3man), 67.86 (Fmoc-CH_2_), 67.69 (C-4man), 66.75 (manO*C*H_2_CH_2_), 63.0 (biotin-NH*C*HCHalkyl), 62.0 (C-6man), 61.07 (biotin-NH*C*HCH_2_S), 56.30 (biotin-NHCH*C*Halkyl), 53.14 (C-α) 47.81 (CH-Fmoc), 40.79 (biotin-CH_2_), 39.89 (manOCH_2_*C*H_2_), 38.45, 37.64 (HN-*C*H_2_-CH_2_-*C*H_2_-NH), 37.59 (C(O)-*C*H_2_-CH_2_-CH_2_-CH_2_-biotin), 36.52 (C-β), 29.29 (HN-CH_2_-*C*H_2_-CH_2_-NH), 29.09 (C(O)-CH_2_-CH_2_-CH_2_-*C*H_2_-biotin), 28.80 (C(O)-CH_2_-CH_2_-*C*H_2_-CH_2_-biotin), 26.27 (C(O)-CH_2_-*C*H_2_-CH_2_-CH_2_-biotin) ppm.

MALDI-TOF-MS: *m/z* = 843.5 [M + H]^+^, 865.4 [M + Na]^+^, 881.4 [M + K]^+^, (842.96 calcd. for C_40_H_54_N_6_O_12_S); ESI-MS: *m/z* = 865.44 [M + Na]^+^ (842.96 calcd. for C_40_H_54_N_6_O_12_S).

#### *N*-(Fluoren-9-ylmethoxycarbonyl)-4-[(+)-biotinylamidopropyl]-1-[2-(α-D-mannopyranosyloxy)ethyl]-L-aspartic acid diamide (10)

The glycoamino acid **8** (250 mg, 0.41 mmol) was dissolved in 80% aq TFA (6 mL) and stirred at RT for 60 min to cleave the *tert*-butyl ester. When the deprotection reaction was complete (TLC control in ethyl acetate:MeOH:H_2_O = 6:2:1) the solvent was removed in vacuo and the residue suspended in MeOH, filtered and the filtrate concentrated under reduced pressure. The resulting crude product was combined with HATU (98.1 mg, 0.28 mmol) and (+)-biotinylamidopropylammonium trifluoroacetate (109 mg, 0.26 mmol), and dried for 30 min under vacuum. DMF (4 mL) and DIPEA (220 μL, 1.65 mmol) were then added under a nitrogen atmosphere. The reaction mixture was stirred overnight at RT, the solvent removed in vacuo and the residue purified by HPLC (MeCN-H_2_O gradient: 0–5 min 100% H_2_O, 5–20 min 60% H_2_O, 20–30 min 40% H_2_O, 30–40 min 0% H_2_O, 40–50 min 0% H_2_O, 50–60 min 100% H_2_O). The title compound was obtained after lyophylisation (90.4 mg, 0.11 mmol, 26%); *R*_f_ = 0.54 (ethyl acetate:MeOH:H_2_O = 6:2:1).

^1^H NMR (600 MHz, CD_3_CN:D_2_O, 1:1): δ = 7.85 (d, 2H, *J* = 7.6 Hz, Fmoc-H), 7.67 (d, 2H, *J* = 7.5 Hz, Fmoc-H), 7.45 (t, 2H, *J* = 7.4 Hz, Fmoc-H), 7.38 (td, 2H, *J* = 7.5 Hz, *J* = 0.9 Hz, Fmoc-H), 4.77 (d, 1H, *J* = 1.7 Hz, H-1man), 4.45 (dd, 1H, *J* = 6.7 Hz, *J* = 4.5 Hz, biotin-NHC*H*CH_2_S), 4.43 (t, 1H, *J* = 6.8 Hz, Hα-asp), 4.40 (dd, 1H, *J* = 12.2 Hz, *J* = 5.5 Hz, Fmoc-C*H*H), 4.37 (dd, 1H, *J* = 12.0 Hz, *J* = 5.3 Hz, Fmoc-CH*H*), 4.29 (dd, 1H, *J* = 5.5 Hz, *J* = 4.4 Hz, CH-Fmoc), 4.27 (dd, 1H, *J* = 6.5 Hz, *J* = 4.7 Hz, biotin-NHC*H*CHalkyl), 3.83 (dd, 1H, *J* = 3.2 Hz, *J* = 1.6 Hz, H-2man), 3.77 (dd, 1H, *J* = 12.2 Hz, J = 6.5 Hz, manOC*H*HCH_2_), 3.69 (dd, 1H, *J* = 9.7 Hz, *J* = 3.2 Hz, H-3man), 3.66 (dd, 1H, *J* = 12.1 Hz, *J* = 6.9 Hz, manOCH*H*CH_2_), 3.64 (dd, 1H, *J* = 12.0 Hz, *J* = 5.3 Hz, H-6man), 3.58 (t, 1H, *J* = 9.8 Hz, H-4man), 3.51 (ddd~dd, 1H, *J* = 9.8 Hz, *J* = 5.5 Hz, H-5man), 3.50 (dd, 1H, *J* = 11.9 Hz, *J* = 5.6 Hz, H-6’man), 3.43 (dd, 1H, *J* = 14.4 Hz, *J* = 6.5 Hz, manOCH_2_CH*H*), 3.33 (dd, 1H, *J* = 13.9 Hz, *J* = 6.1 Hz, manOCH_2_C*H*H), 3.17 (td, 1H, *J* = 7.1 Hz, *J* = 6.9 Hz, biotin-NHCHC*H*alkyl), 3.12, 3.10 (each dd, each 1H, HN-C*H*_2_-CH_2_-CH_2_-NH), 3.09, 3.07 (each dd, each 1H, NH(C=O)C*H*_2_-CH_2_-CH_2_-CH_2_-biotin), 2.66, 2.54 (each dd, each 1H, HN-CH_2_-CH_2_-C*H*_2_-NH), 2.11, 2.09 (each dd, each 1H, 2Hβ-asp), 1.61, 1.58 (each dd, each 1H, HN-CH_2_-C*H*_2_-CH_2_-NH), 1.52 (dd, 1H, *J* = 13.9 Hz, *J* = 6.7 Hz, NH(C=O)CH_2_-CH_2_-CH_2_-C*H*H-biotin), 1.50 (dd, 1H, *J* = 14.0 Hz, *J* = 6.8 Hz, NH(C=O)CH_2_-CH_2_-C*H*H-CH_2_-biotin), 1.48 (dd, 1H, *J* = 13.9 Hz, *J* = 7.2 Hz, NH(C=O)CH_2_-CH_2_-CH_2_-CH*H*-biotin), 1.45 (dd, 1H, *J* = 14.2 Hz, *J* = 7.0 Hz, NH(C=O)CH_2_-CH_2_-CH*H*-CH_2_-biotin), 1.30, 1.27 (each dd, each 1H, NH(C=O)CH_2_-C*H*_2_-CH_2_-CH_2_-biotin) ppm; ^13^C NMR (150.92 MHz, CD_3_CN:D_2_O = 1:1): δ = 175.93 (NH-(C=O)_asp_), 172.84 (NH-*C*(O)-(CH_2_)_4_-biotin), 171.53 (C-γ), 165.08 ((NH_2_)_2_C=O), 157.25 (Fmoc-C=O), 144.24, 144.19, 129.51, 128.51, 128.40, 127.83 (aryl-Fmoc), 100.1 (C-1man), 73.22 (C-5man), 71.12 (C-3man), 68.07 (C-2man), 67.38 (Fmoc-CH_2_), 67.13 (C-4man), 66.01 (C-6man), 62.25 (biotin-NH*C*HCHalkyl), 61.36 (manO*C*H_2_CH_2_), 60.50 (biotin-NH*C*HCH_2_S), 57.67 (biotin-NHCH*C*Halkyl), 52.61 (C-α), 47.22 (Fmoc-CH), 39.35 (biotin-NHCH*C*H_2_S), 38.36 (manOCH_2_*C*H_2_), 37.07, 36.84 (HN-*C*H_2_-CH_2_-*C*H_2_-NH), 35.95 (C(O)-*C*H_2_-CH_2_-CH_2_-CH_2_-biotin), 28.69 (C-β), 28.52 (HN-CH_2_-*C*H_2_-CH_2_-NH), 28.22 (C(O)-CH_2_-CH_2_-CH_2_-*C*H_2_-biotin), 25.66 (C(O)-CH_2_-CH_2_-*C*H_2_-CH_2_-biotin), 25.53 (C(O)-CH_2_-*C*H_2_-CH_2_-CH_2_-biotin) ppm.

MALDI-TOF-MS: *m/z* = 865.3 [M + Na]^+^; ESI-MS: *m/z* = 865.34 [M + Na]^+^ (842.96 calcd. for C_40_H_54_N_6_O_12_S).

#### *N*-[*p*-(Trifluoromethyl-diazirinyl)-benzoyl]-4-[2-(α-D-mannopyranosyloxy)ethyl]-1-[(+)-biotinylamidopropyl]-L-aspartic acid diamide (12)

The glycoamino acid derivative **9** (25.0 mg, 29.7 μmol) was dissolved in piperidine (20% in DMF, 3.0 mL) and stirred at RT for 60 min to remove the Fmoc protecting group. The solvent was removed in vacuo and the resulting crude product combined with HATU (11.3 mg, 29.7 μmol) and the diazirine **11** (7.2 mg, 31.0 μmol), and dry DMF (1.5 mL) added under a nitrogen atmosphere. DIPEA (0.03 mL, 87.0 μmol) was then added and the reaction mixture stirred overnight at RT. The solvent was removed under reduced pressure and the residue purified by flash chromatography with the exclusion of light (ethyl acetate:MeOH:H_2_O = 6:2:1) to yield, after lyophilisation, the title compound (20.1 mg, 24.1 μmol, 81%); *R*_f_ = 0.51; [α]_D_^20^ +23.0 (*c* 0.12 mM, MeCN:H_2_O = 1:1); UV–Vis (*c* 0.12 mM, MeCN:H_2_O = 1:1): λ_max_(1) = 353.3 nm, ε(1) = 6000 Lmol^−1^cm^−1^; λ_max_(2) = 303.5 nm, ε(2) = 10000 Lmol^−1^cm^−1^; λ_max_(3) = 285.2 nm, ε(3) = 24000 Lmol^−1^cm^−1^; λ_max_(4) = 251.6 nm, ε(4) = 37000 Lmol^−1^cm^−1^; FT-IR (KBr): 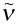 = 3447.5 cm^−1^, 2928.6 cm^−1^, 2381.0 cm^−1^, 1654.2 cm^−1^, 1560.1 cm^−1^, 1267.9 cm^−1^, 1136.9 cm^−1^, 1101.9 cm^−1^, 1059.5 cm^−1^, 767.9 cm^−1^.

^1^H NMR (600 MHz, CD_3_CN:D_2_O = 1:1): δ = 7.90 (dt, 2H, *J* = 8.7 Hz, *J* = 2.0 Hz, aryl-H), 7.39 (d, 2H, *J* = 8.2 Hz, aryl-H), 4.81 (dd, 1H, *J* = 7.7 Hz, *J* = 6.1 Hz, Hα-asp), 4.75 (d, 1H, *J* = 1.6 Hz, H-1man), 4.50 (dd, 1H, *J* = 7.9 Hz, *J* = 4.9 Hz, biotin-NH_2_C*H*CHalkyl), 4.31 (dd, 1H, *J* = 7.9 Hz, *J* = 4.5 Hz, biotin-NH_2_C*H*CH_2_S), 3.82 (dd, 1H, *J* = 3.4 Hz, *J* = 1.7 Hz, H-2man), 3.76 (dd, 1H, *J* = 12.1 Hz, *J* = 2.2 Hz, manOC*H*HCH_2_), 3.69 (dd, 1H, *J* = 9.3 Hz, *J* = 3.4 Hz, H-3man), 3.67 (dd, 1H, *J* = 12.0 Hz, *J* = 2.4 Hz, manOCH*H*CH_2_), 3.58 (t, 1H, *J* = 9.8 Hz, H-4man), 3.52 (ddd, 1H, *J* = 9.8 Hz, *J* = 5.3 Hz, *J* = 2.2 Hz, H-5man), 3.49 (dd, 1H, *J* = 5.5 Hz, *J* = 4.2 Hz, H-6man), 3.42 (dd, 1H, *J* = 5.7 Hz, *J* = 2.3 Hz, H-6’man), 3.39 (dd, 1H, *J* = 6.7 Hz, *J* = 12.3 Hz, manOCH_2_CH*H*), 3.30 (dd, 1H, *J* = 6.4 Hz, *J* = 12.3 Hz, manOCH_2_C*H*H), 3.27, 3.24 (each dd, each 1H, HN-CH_2_-CH_2_-C*H*_2_-NH), 3.20 (dd, 1H, *J* = 7.7 Hz, *J* = 6.8 Hz, biotin-NH_2_CHC*H*alkyl), 3.13, 3.12 (each dd, each 1H, NH(C=O)C*H*_2_-CH_2_-CH_2_-CH_2_-biotin), 2.91 (dd, 1H, *J* = 13.0 Hz, *J* = 5.0 Hz, biotin-*NH_2_*CHC*H*H*S*), 2.82, 2.72 (each dd, each 1H, HN-C*H*_2_-CH_2_-CH_2_-NH), 2.70 (dd, 1H, *J* = 13.1 Hz, *J* = 6.4 Hz, biotin-NH_2_CHCH*H*S), 2.16, 2.15 (each dd, each 1H, NH(C=O)CH_2_-CH_2_-CH_2_-C*H*_2_-biotin), 1.68, 1.67 (each dd, each 1H, NH(C=O)CH_2_-CH_2_-C*H*_2_-CH_2_-biotin), 1.63 (dd, 1H, *J* = 13.3 Hz, *J* = 6.7 Hz, NH(C=O)CH_2_-C*H*H-CH_2_-CH_2_-biotin), 1.57 (dd, 1H, *J* = 15.1 Hz, *J* = 7.4 Hz, HN-C*H*_2_-C*H*H-CH_2_-NH), 1.53 (dd, 1H, *J* = 13.1 Hz, *J* = 6.9 Hz, NH(C=O)CH_2_-CH*H*-CH_2_-CH_2_-biotin), 1.51 (dd, 1H, *J* = 13.9 Hz, *J* = 6.8 Hz, Hβ-asp), 1.36 (dd, 1H, *J* = 15.5 Hz, *J* = 7.2 Hz, HN-C*H*_2_-CH*H*-CH_2_-NH), 1.30 (dd, 1H, *J* = 13.4 Hz, *J* = 6.9 Hz, Hβ’-asp) ppm; ^13^C NMR (150.90 MHz, CD_3_CN:D_2_O = 1:1): δ = 176.60 (aryl-C=O), 172.78 (NH-(C=O)_asp_), 172.37 (C-γ), 168.61 (NH-*C*(O)-CH_2_-(CH_2_)_3_-biotin), 165.66 ((NH_2_)_2_C=O), 135.57, 133.14, 129.11, 127.73 (aryl-C), 124.44 (q, *J_C,F_* = Hz, CF_3_), 100.72 (C-1man), 87.77 (N=N-*C*CF_3_) 73.82 (C-5man), 71.68 (C-3man), 71.10 (C-2man), 67.70 (C-4man), 66.73 (C-6man), 62.81 (biotin-NH_2_*C*HCHalkyl), 61.95 (manO*C*H_2_CH_2_), 61.09 (biotin-NH_2_*C*HCH_2_S), 56.29 (biotin-NH_2_CH*C*Halkyl), 52.36 (C-α), 40.78 (biotin-NH_2_CH*C*H_2_S), 39.89 (manOCH_2_*C*H_2_), 38.13 (HN-*C*H_2_-CH_2_-CH_2_-NH), 37.68 (HN-CH_2_-CH_2_-*C*H_2_-NH), 37.36 (NH-C(O)-*C*H_2_-(CH_2_)_3_-biotin), 36.49 (C-β), 29.71 (HN-CH_2_-*C*H_2_-CH_2_-NH), 29.24 (NH-C(O)-(CH_2_)_3_-*C*H_2_-biotin), 28.78 (NH-C(O)-CH_2_-CH_2_-*C*H_2_-CH_2_-biotin), 26.24 (NH-C(O)-CH_2_-*C*H_2_-CH_2_-CH_2_-biotin) ppm.

MALDI-TOF-MS: *m/z* = 827.3 [M – N_2_ + Na]^+^; ESI-MS: *m/z* = 855.29 [M + Na]^+^, 827.29 [M – N_2_ + Na]^+^ (832.85 calcd. for C_34_H_47_F_3_N_8_O_11_S).

#### *N*-[*p*-(Trifluoromethyl-diazirinyl)-benzoyl]-4-[(+)-biotinylamidopropyl]-1-[2-(α-D-mannopyranosyloxy)ethyl]-L-aspartic acid diamide (13)

The glycoamino acid derivative **10** (19.6 mg, 23.2 μmol) was dissolved in piperidine (20% in DMF, 1.5 mL) and stirred at RT for 60 min to remove the Fmoc protecting group. The solvent was removed in vacuo and the resulting crude product combined with HATU (14.0 mg, 36.8 μmol) and the diazirine **11** (7.2 mg, 30.9 μmol), and dry DMF (1.5 mL) added under a nitrogen atmosphere. DIPEA (0.05 mL, 145 μmol) was then added and the reaction mixture stirred overnight at RT. The solvent was removed under reduced pressure and the residue purified by flash chromatography with the exclusion of light (ethyl acetate:MeOH:H_2_O = 6:2:1) to yield, after lyophilisation, the title compound (19 mg, 22.8 μmol, 98%); *R*_f_ = 0.33; [α]_D_^20^ +30.0 (*c* 0.12 mM, MeCN:H_2_O = 1:1); UV-Vis (*c* 0.12 mM, MeCN:H_2_O = 1:1): λ_max_(1) = 335.9 nm, ε(1) = 9833.3 Lmol^−1^cm^−1^; λ_max_(2) = 278.2 nm, ε(2) = 18333.3 Lmol^−1^cm^−1^; λ_max_(3) = 233.5 nm, ε(3) = 23333.3 Lmol^−1^cm^−1^; FT-IR (KBr): 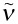 = 3447.7 cm^−1^, 2940.5 cm^−1^, 2380.9 cm^−1^, 1684.7 cm^−1^, 1654.2 cm^−1^, 1636.6 cm^−1^, 1560.1 cm^−1^, 1438.3 cm^−1^, 1400.2 cm^−1^, 1279.8 cm^−1^, 1202.4 cm^−1^, 1113.1 cm^−1^, 761.9 cm^−1^.

^1^H NMR **(**600 MHz, CD_3_CN-D_2_O, 1:1): δ = 8.23 (dd, 2H, *J* = 8.5 Hz, *J* = 1.4 Hz, aryl-H), 7.38 (dd, 2H, *J* = 8.8 Hz, *J* = 1.3 Hz, aryl-H), 4.86 (dd, 1H, *J* = 8.6 Hz, *J* = 5.3 Hz, Hα-asp), 4.76 (d, 1H, *J* = 1.5 Hz, H-1man), 4.49 (dd, 1H, *J* = 7.7 Hz, *J* = 4.4 Hz, biotin-NHC*H*CH_2_S), 4.30 (dd, 1H, *J* = 8.0 Hz, *J* = 4.4 Hz, biotin-NHC*H*CHalkyl), 3.80 (dd, 1H, *J* = 3.3 Hz, *J* = 1.7 Hz, H-2man), 3.73 (dd, 1H, *J* = 12.2 Hz, *J* = 2.3 Hz, manOC*H*HCH_2_), 3.69 (dd, 1H, *J* = 11.0 Hz, *J* = 4.3 Hz, H-6man), 3.68 (dd, 1H, *J* = 9.7 Hz, *J* = 3.2 Hz, H-3man), 3.66 (dd, 1H, *J* = 12.2 Hz, *J* = 3.3 Hz, manOCH*H*CH_2_), 3.59 (t, 1H, *J* = 9.8 Hz, H-4man), 3.52 (dd, 1H, *J* = 10.6 Hz, *J* = 4.4 Hz, H-6’man), 3.49 (dd, 1H, *J* = 10.0 Hz, *J* = 4.1 Hz, H-5man), 3.44 (dd, 1H, *J* = 6.8 Hz, *J* = 4.1 Hz, manOCH_2_C*H*H), 3.32 (dd, 1H, *J* = 6.1 Hz, *J* = 4.0 Hz, manOCH_2_CH*H*), 3.20 (dd, 1H, *J* = 8.8 Hz, *J* = 5.4 Hz, biotin-NHCHC*H*alkyl), 3.19 (dd, 1H, *J* = 13.7 Hz, *J* = 6.9 Hz, HN-C*H*H-CH_2_-CH_2_-NH), 3.16, 3.10 (each dd, each 1H, NH(C=O)C*H*_2_-CH_2_-CH_2_-CH_2_-biotin), 3.05 (dd, 1H *J* = 13.4 Hz, *J* = 7.0 Hz, HN-CH*H*-CH_2_-CH_2_-NH), 2.90 (dd, 1H, *J* = 13.0 Hz, *J* = 5.1 Hz, biotin-NHCHCH*H*S), 2.77 (dd, 1H, *J* = 14.7 Hz, *J* = 5.3 Hz, HN-CH_2_-CH_2_-C*H*H-NH), 2.69 (dd, 1H, *J* = 12.6 Hz, *J* = 4.8 Hz, biotin-NHCHC*H*HS), 2.68 (dd, 1, *J* = 14.9 Hz, *J* = 5.8 Hz, HN-CH_2_-CH_2_-CH*H*-NH), 2.16 (dd, 1H, *J* = 15.3 Hz, *J* = 7.7 Hz, Hβ-asp), 2.12 (dd, 1H, *J* = 14.9 Hz, *J* = 7.5 Hz, Hβ’-asp), 1.66 (ddd, 1H, *J* = 13.8 Hz, *J* = 7.1 Hz, *J* = 6.2 Hz, NH(C=O)CH_2_-CH*H*-CH_2_-CH_2_-biotin), 1.64 (dd, 1H, *J* = 15.3 Hz, *J* = 6.2 Hz, NH(C=O)CH_2_-CH_2_-CH*H*-CH_2_-biotin), 1.57 (ddd, 1H, *J* = 13.3 Hz, *J* = 6.8 Hz, *J* = 6.3 Hz, NH(C=O)CH_2_-C*H*H-CH_2_-CH_2_-biotin), 1.55 (dd, 1H, *J* = 15.5 Hz, *J* = 6.8 Hz, NH(C=O)CH_2_-CH_2_-C*H*H-CH_2_-biotin), 1.53, 1.52 (each dd, each 1H, NH(C=O)CH_2_-CH_2_-CH_2_-C*H*_2_-biotin), 1.34, 1.27 (each dd, each 1H, *J* = 15.3 Hz, *J* = 7.0 Hz, HN-CH_2_-C*H*_2_-CH_2_-NH) ppm; ^13^C NMR (150.90 MHz, CD_3_CN:D_2_O = 1:1): δ = 176.86 (C-γ), 172.58 (NH-(C=O)_asp_), 171.47 (NH(*C*=O)-(CH_2_)_4_-biotin), 168.28 (aryl-C=O), 165.16 ((NH_2_)_2_C=O), 149.11, 139.43 (aryl-C), 136.19 (q, *J_C,F_* = Hz, CF_3_), 129.08, 128.99 (aryl-C), 100.67 (C-1man), 84.21 (N=N-*C*CF_3_), 73.78 (C-5man), 71.69 (C-2man), 71.12 (C-3man), 67.74 (C-4man), 66.57 (C-6man), 62.85 (biotin-NH*C*HCHalkyl), 61.92 (manO*C*H_2_CH_2_), 61.09 (biotin-NH*C*HCH_2_S), 56.32 (biotin-NHCH*C*Halkyl), 52.34 (C-α), 40.78 (biotin-NHCH*C*H_2_S), 39.97 (manOCH_2_*C*H_2_), 38.47, 37.63 (HN-*C*H_2_-CH_2_-*C*H_2_-NH), 37.39 (NH(C=O)*C*H_2_-(CH_2_)_3_-biotin), 36.50 (C-β), 29.21 (C- HN-CH_2_-*C*H_2_-CH_2_-NH), 29.08 (NH(C=O)-(CH_2_)_3_-*C*H_2_-biotin), 28.79, 26.23 (NH(C=O)-CH_2_-*C*H_2_-*C*H_2_-CH_2_-biotin) ppm.

MALDI-TOF-MS: *m/z* = 855.3 [M + Na]^+^; ESI-MS: *m/z* = 827.31 [M – N_2_ + Na]^+^; 855.31 [M + Na]^+^ (832.85 calcd. for C_34_H_47_F_3_N_8_O_11_S).
